# 966. ID Fellows Cup: Leveraging Gamification and Social Media to Enhance Clinical Infectious Diseases Education

**DOI:** 10.1093/ofid/ofab466.1161

**Published:** 2021-12-04

**Authors:** Lauren Nicholas Herrera, Nathan Nolan, Miguel A Chavez, Mauricio J Kahn, John D Cleveland, Todd P McCarty, Gerome Escota, Prathit A Kulkarni, Mukesh Patel, Jorge M Rodriguez, Hillary P Hunsinger, Donald Dempsey, James Willig, Jeremey Walker

**Affiliations:** 1 University of Alabama at Birmingham, Birmingham, Alabama; 2 Washington U Sch of Med, St. Louis, Missouri; 3 Barnes Jewish Hospital, St. Louis, Missouri; 4 University of Alabama at Birmingham; Birmingham VA Medical Center, Birmingham, Alabama; 5 Washington University School of Medicine; 6 Baylor College of Medicine / Michael E. DeBakey VA Medical Center, Houston, TX; 7 UAB, Birmingham, Alabama; 8 University of Arizona College of Medicine, Tucson, Arizona; 9 University of Alabama in Birmingham, Birmingham, AL

## Abstract

**Background:**

We hypothesized that we could leverage social media to recruit learners to a gamification-infused ID knowledge competition, and entice them to explore additional online educational resources.

**Methods:**

We created the ID Fellows Cup, a knowledge-based trivia competition, to engage Infectious Diseases fellows. The game was crafted via Kaizen-Education, a software platform developed at the University of Alabama at Birmingham, that uses gamification to engage learners. Multiple choice questions including figures and/or text are presented to learners, followed by detailed teaching explanations. 60 questions emphasizing high-yield concepts were delivered over 4 weeks. Questions were written by fellows and reviewed by faculty at three programs. Elements of gamification (virtual rewards, leaderboards, etc.) were included to enhance engagement. Recruitment strategies included Twitter, program director emails, and peer-to-peer. We measured game statistics and participation. Learners were invited to complete a post-game survey about their experience.

**Results:**

Table 1 shows our game statistics with broad geographic reach including 42 programs. Most fellows matriculated in 2019 or 2020; the number of US ID fellows equaled 17% of those completing ID in-training exam. Recruitment sources included 44% co-fellow, 42% Twitter, and 15% Program Director. Through 20 days with questions, we had 155 daily average users. Overall, fellows answered 11,419 total questions, representing 89% of all released questions. Of 103 responses to post-game survey (table 2) 97% would participate again and all felt the game was a good use of their time. Over 80% of participants reported some engagement with linked resources included in the answer explanations. In general, 78% felt engagement with online resources increased subsequent to participating in the game, including learning about at least one new online resource.

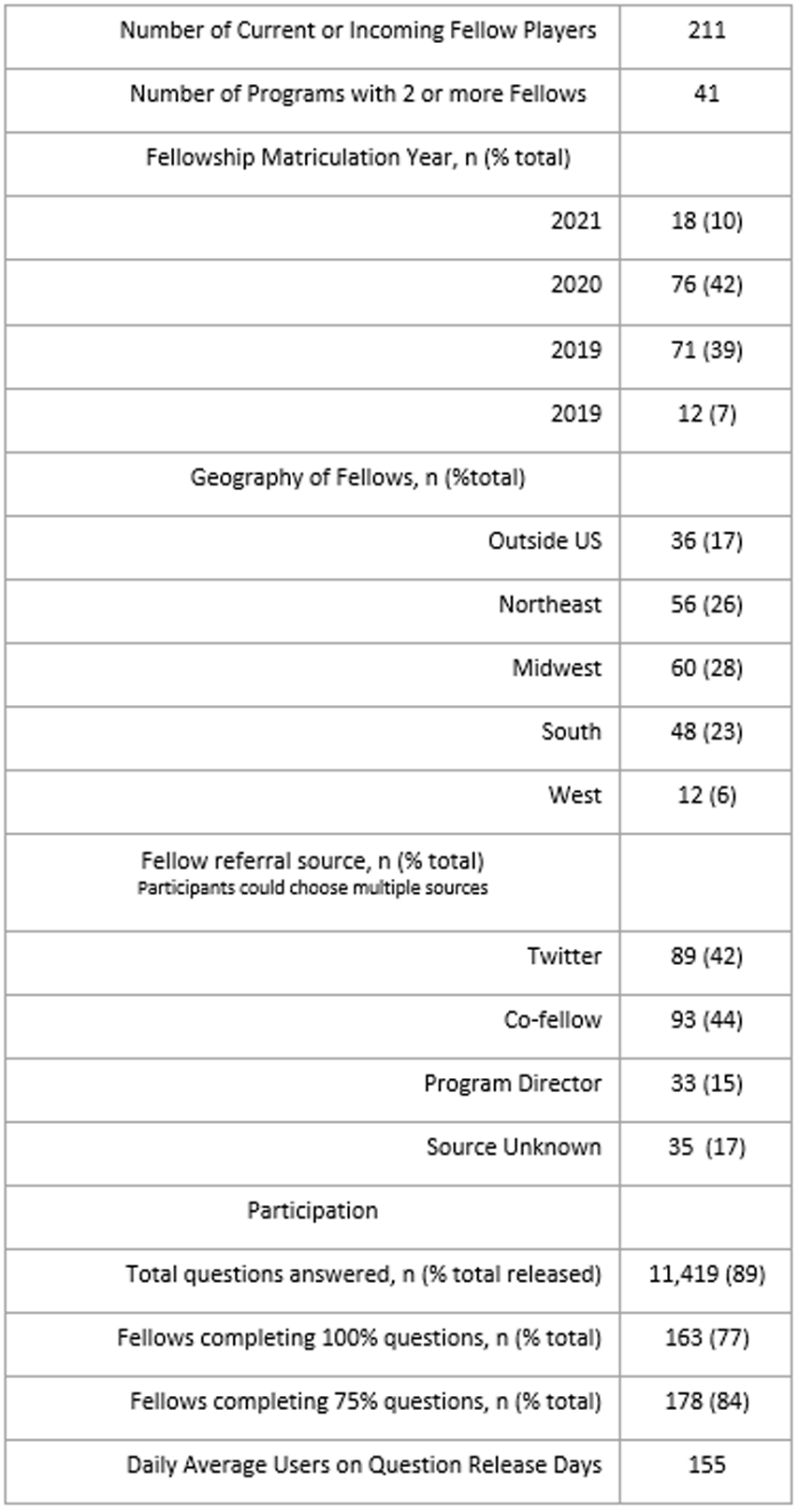

**Conclusion:**

We leveraged social media and gamification to effectively engage, and stimulate ID learners to explore additional online educational resources. Technology enriched learning, helps supplement and globalize ID education, making it as diverse and engaging as our field.

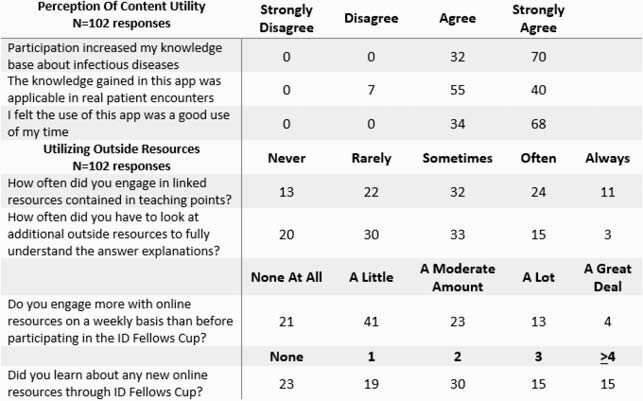

**Disclosures:**

**Todd P. McCarty, MD**, **Cidara** (Grant/Research Support)**GenMark** (Grant/Research Support, Other Financial or Material Support, Honoraria for Research Presentation)**T2 Biosystems** (Consultant) **Prathit A. Kulkarni, M.D.**, **Vessel Health, Inc.** (Grant/Research Support)

